# Hair Follicle Dermal Sheath Derived Cells Improve Islet Allograft Survival without Systemic Immunosuppression

**DOI:** 10.1155/2015/607328

**Published:** 2015-04-27

**Authors:** Xiaojie Wang, Jianqiang Hao, Gigi Leung, Trisia Breitkopf, Eddy Wang, Nicole Kwong, Noushin Akhoundsadegh, Garth L. Warnock, Jerry Shapiro, Kevin J. McElwee

**Affiliations:** ^1^Department of Dermatology and Skin Science, University of British Columbia, 835 West 10th Avenue, Vancouver, BC, Canada V5Z 4E3; ^2^Department of Surgery, University of British Columbia, 950 West 10th Avenue, Vancouver, BC, Canada V5Z 1M9

## Abstract

Immunosuppressive drugs successfully prevent rejection of islet allografts in the treatment of type I diabetes. However, the drugs also suppress systemic immunity increasing the risk of opportunistic infection and cancer development in allograft recipients. In this study, we investigated a new treatment for autoimmune diabetes using naturally immune privileged, hair follicle derived, autologous cells to provide localized immune protection of islet allotransplants. Islets from Balb/c mouse donors were cotransplanted with syngeneic hair follicle dermal sheath cup cells (DSCC, group 1) or fibroblasts (FB, group 2) under the kidney capsule of immune-competent, streptozotocin induced, diabetic C57BL/6 recipients. Group 1 allografts survived significantly longer than group 2 (32.2 ± 12.2 versus 14.1 ± 3.3 days, *P* < 0.001) without administration of any systemic immunosuppressive agents. DSCC reduced T cell activation in the renal lymph node, prevented graft infiltrates, modulated inflammatory chemokine and cytokine profiles, and preserved better beta cell function in the islet allografts, but no systemic immunosuppression was observed. In summary, DSCC prolong islet allograft survival without systemic immunosuppression by local modulation of alloimmune responses, enhancing of beta cell survival, and promoting of graft revascularization. This novel finding demonstrates the capacity of easily accessible hair follicle cells to be used as local immunosuppression agents in islet transplantation.

## 1. Introduction

Transplantation of pancreatic islets is potentially a curative treatment for type 1 diabetes. However, the systemic immunosuppressive drugs that recipients must use lifelong to prevent rejection of the islet allografts also suppress immunity to other antigens, thereby increasing the risk of opportunistic infections and cancers [1–3]. These drugs also have an adverse impact on the transplanted islets' survival, causing the function of islets to decline over time, such that many recipients must eventually resume insulin injections for control of blood glucose levels [1–3]. Therefore, a safe and efficient means to protect graft rejection is urgently needed.

Localized immune protection is a feasible means to provide an immune privileged microenvironment to prevent rejection with minimal systemic side effects [4–7]. It has been reported that allogeneic islets could survive in the anterior chamber of the eye [4]; however, to date, corneal transplantation is the only common clinical procedure that takes advantage of natural immune privilege (IP), due to the feasibility of transplantation from and to the same IP site. Although it seems unlikely that islets could be transplanted into natural IP sites and remain functional in practice, the survival of ectopic IP cells/tissues has led to a novel idea that they could be used in localized, cell-based therapy.

Recent progress using cells with IP properties has explored the potential of cell-based immune modulation as an alternative to immunosuppressive drug therapy in the context of pancreatic islet transplantation [4–8]. Strategies using cells with natural IP properties, such as amniotic epithelial cells or Sertoli cells, derived from the placenta and testis, respectively, have been successfully cotransplanted with isolated islets. Cotransplantation of either amniotic epithelial cells or Sertoli cells sustains islet allograft survival, without systemic immunosuppression, suggesting that a more physiological approach to immune protection of transplanted islets can be achieved. However, the supply of these cells is limited; they are difficult to derive and to maintain in long-term culture.

The hair follicle (HF) constitutes one of the few immunologically privileged tissues besides the brain, eye, placenta, and testis [9–12]. Hair follicle IP has been demonstrated in fully MHC mismatched donor and recipient HF tissue transplantation both in mouse and human studies [10, 13]. New hair was produced in recipients transplanted with the lower parts of HFs from a histoincompatible donor, suggesting that HFs can escape alloimmune attack [10]. Studies into the mechanisms of alopecia areata and lichen planopilaris hair loss reveal that normal hair follicles may avoid autoimmune mediated destruction by downregulating MHC I and II and by upregulating potent immunosuppressive factors [9, 12]. Our group has previously shown that primary HF bulb and cultured dermal sheath cup cells (DSCC) exhibit immune privilege via somatostatin and PD-L1 expression, respectively [13–15].

Hair follicle (HF) cells with IP are readily accessible and they can be derived from HFs of the islet transplant recipient (autologous), suggesting their potential use in personalized immunoprotective transplant medicine. In this study, we examined the effects of cultured DSCC on beta cell survival in a mouse islet transplantation model.

## 2. Research Design and Methods

### 2.1. Islet Isolation and Transplantation

C57BL/6 (B6), C3H/HeJ (C3H), and Balb/c mice were purchased from The Jackson Laboratory.   All mice were cared for according to the guidelines of the Canadian Council on Animal Care and regulations of the University of British Columbia. Donor islets were isolated by ductal collagenase injection from 8–10-week-old female Balb/c mice [16]. Three groups of 400 islets each were transplanted into the left kidney of 200 mg/kg streptozotocin (Sigma, Oakville, Canada) induced diabetic age-matched recipients one to two weeks prior to transplantation.

400 islets were either mixed with DSCC (group 1) or with dermal fibroblasts (group 2) in a collagen gel [17], which contained 350 *μ*L 3 × HAM's F10 medium, 26 *μ*L 0.4 N NaOH, 125 *μ*L FBS, and 870 *μ*L of acid-extracted fetal bovine type-I collagen (5 mg/mL, Sigma), plus DSCC or FB (1 × 10^5^ per graft). Animals were considered as diabetic after two consecutive days with random blood glucose levels >20 mmol/L. Allograft function in transplanted mice was defined as a drop of blood glucose concentration <13.8 mmol/L on day 3 after transplantation, and a graft was considered as rejected when the blood glucose rebounded to a level of >13.8 mmol/L for two consecutive days after normal primary graft success.

### 2.2. Microdissection of HF Cells

Dermal sheath cup (DSC), dermal papilla (DP), nonbulbar dermal sheath (DS) tissues, and nonfollicular dermal fibroblasts (FB) were isolated from donor mouse vibrissa HFs under a microscope as described [13–15]. The bulb mesenchyme tissue was cut to separate the lowest portion of bulbar dermal sheath cup (DSC) and the DP. The DS is defined as the sheath surrounding the HF that extends from above the bulb region to below the sebaceous gland duct. The individual tissues were cultured in AmnioMax C-100 with supplement (Invitrogen, Burlington, ON).

### 2.3. Glucose Stimulation Assay

The isolated islets were cultured with DSCC or FB cells in Krebs-Ringer's Buffer (Sigma) at 37°C for 1 hr after 3 or 7 days of coculture. The supernatant was then replaced with Krebs-Ringer's Buffer with 2 mmol/L or 20 mmol/L of glucose and cultured for 1 hr and stored for insulin detection. Insulin was measured by ELISA (Crystal Chem., Chicago, IL).

### 2.4. Mixed-Lymphocyte Reaction Assay (MLR)

The responder splenocytes (SPL) or graft draining renal lymph node (LN) derived cells were isolated from B6 recipients. Responder cells were stimulated with *γ*-irradiated (2500 rad) Balb/c (allogeneic) or C3H (third party) derived splenocytes at various stimulator/responder ratios for 4 days. BrdU enzyme-linked immunosorbent assay (ELISA; cat#ab126556, Abcam, Toronto, ON, Canada) was performed according to the manufacturer's instructions. Briefly, the cells were labeled with BrdU for 16 h, followed by fix/denature procedures. The incorporated BrdU was detected at 450 nm after incubation with anti-BrdU peroxidase. IL-2 secretion was detected by using antibody pairs (capture mAb: clone JES6-1A12; detection mAb: clone JES6-5H4) and recombinant protein standard (cat#39-8021) (eBioscience, San Diego, CA).

### 2.5. Histological Analysis

The transplanted grafts were removed under a microscope and fixed in 4% paraformaldehyde. Paraffin sections (5 *μ*m) were stained with hematoxylin and eosin (H&E). Staining of insulin (DAKO, Burlington, ON, Canada) and CD45 (R&D, Burlington, ON, Canada) was developed using DAB (Sigma).

### 2.6. Flow Cytometric Analysis

Single-cell suspensions were stained with fluorochrome-conjugated mAbs CD4, CD25, Foxp3, CD69, and CD44 (all eBioscience). Intracellular staining of cytokine IFN*γ* and IL-2 (eBioscience) was performed after 4 to 5 h phorbol myristate acetate (PMA) plus ionomycin (Sigma) stimulation.

### 2.7. Quantitative RT-PCR

Total RNA from cultured DSCC, DP, DS, and FB or cultured islets or allografts were extracted by using the RNeasy Mini Kit (Qiagen, Mississauga, Canada) with its on-column RNase-free DNase I procedure. cDNA was then synthesized by Superscript III Reverse Transcriptase (Invitrogen) in 20 *μ*L reaction including 0.5 *μ*g RNA and 150 ng random primer. RNaseH was utilized to remove complementary RNA. QPCR was performed in duplicate using SYBR Green (Applied Biosystems, Carlsbad, CA) in a 10 *μ*L volume containing 1 ng/*μ*L of cDNA and 0.4 *μ*M of each primer (IDT, Toronto, ON, Canada) (Table 1). Relative expression level was expressed as 2^−(CT18s-CTgene)^ (where CT is cycling threshold) with 18S RNA as the endogenous control for normalization.

### 2.8. Western Blot

30 to 50 *μ*g of each cell lysate from cultured DSCC or FB (*n* = 3) was loaded onto a 12% separating Bis-Tris gel. The proteins were transferred to a nitrocellulose membrane (BioRad, Mississauga, Canada). The membrane was blocked in PBS containing 5% skim milk for 1 h followed by incubation with the primary antibody rabbit anti-mouse BMP6 (ab15640), Inhibin beta A (22689-1-AP), Fgf2 (sc-79, Santa Cruz, Dallas, TX), or mouse anti-actin. The blot was developed with Enhanced Chemiluminescence Plus Developer (Pierce, Nepean, ON, Canada). The protein expression level was calculated using ImageJ software [18].

### 2.9. Statistical Analysis

The significance of the Kaplan-Meier survival curve (Figure 2) was determined by log-rank test using Prism software (http://www.prismmodelchecker.org/). The significance of T cell proliferation, expression of T cell subsets, and cytokine production was calculated by two-tailed Student *t*-test or ANOVA. Values are expressed as means ± SEM, and differences are considered significant when *P* < 0.05.

## 3. Results

### 3.1. DSCC Stimulates Reduced Alloimmune Responses and Promotes Beta Cell Survival* In Vitro*

**Figure 1:**
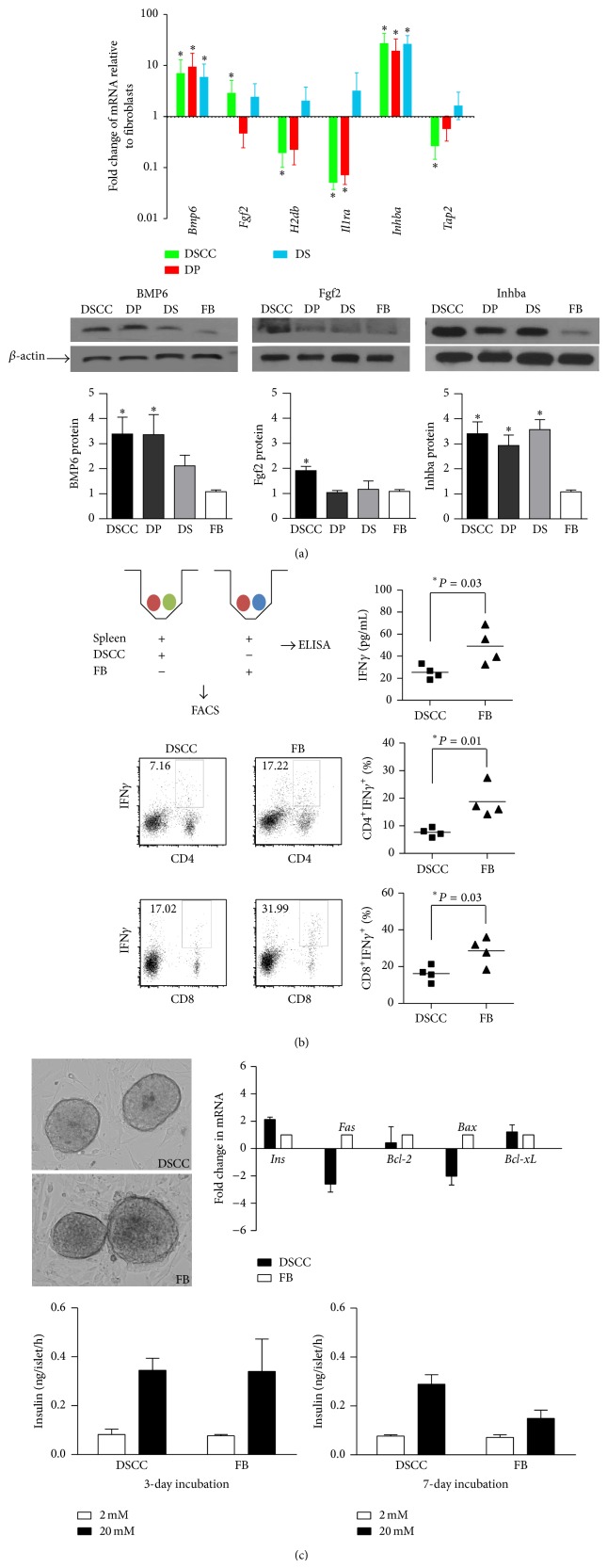
DSCC stimulates reduced alloimmune responses and promotes beta cell survival* in vitro*. (a) Immune privilege related genes were expressed in DSCC. Hair follicle dermal sheath cup cells (DSCC), dermal papilla (DP), and dermal sheath (DS) cell gene expression were examined relative to nonfollicular fibroblasts (FB). Values were obtained using the 2^−ΔΔCT^ fold change method. For standard error (SE), the standard deviation of relevant samples was divided by the square root of the number of samples. The calculation for error bars was 2^−ΔΔCT + SE^ minus fold change or fold change minus 2^−ΔΔCT − SE^ to find the range of difference. The fold change was generated from duplicated values (*n* = 3). (b) DSCC significantly inhibited allogeneic leukocyte IFN*γ* secretion relative to FB. DSCC or FB from B6 (2 × 10^4^) were cocultured with splenic leukocytes (Balb/c, 2 × 10^5^) for 5 days. IFN*γ* detected in supernatant was significantly less in the presence of DSCC than FB. Data are shown as mean pg/mL IFN*γ* of three different experiments ± SEM, with Student *t*-test showing significance, ^∗^ *P* < 0.05. Percentage of IFN*γ* expressing cells in CD4^+^ or CD8^+^ subsets after 5 days of coculture was presented by flow cytometric analysis. Representative dot plots and accumulated data from CD4^+^IFN*γ* ^+^ or CD8^+^IFN*γ* ^+^ cell populations are shown. (c) DSCC preserved beta cell function. DSCC or FB from B6 mice were cocultured with islets from Balb/c mice; the ability of insulin secretion was measured by static incubation at 3 and 7 days and relative gene expression was detected by qPCR at day 7. A similar insulin secretion upon glucose stimulation was detected at day 3. However, better insulin secretion was observed at day 7 with 20 mM glucose stimulation. Increased insulin mRNA and decreased* Fas* and* Bax* mRNA gene expression were detected at culture day 7. BMP6: bone morphogenetic protein 6; Fgf2: fibroblast growth factor 2; Il1ra: interleukin 1 receptor antagonist; Inhba: Inhibin beta A; Tap2: transporter associated with antigen processing. Data represent 3 independent experiments and are expressed as means ± SEM. Statistical significance was determined by Student *t*-test, ^∗^ *P* < 0.05.

We proposed to use cultured HF derived IP cells to protect islet allograft survival. Therefore, we first examined IP-related gene expression in DSCC, DP, and DS cells cultured for 4 passages, relative to FB, by qPCR (Figure 1(a)). Decreased* MHC I* (*H2db*),* Tap2*, and* Il1ra* and increased* Inhba* were identified in DSCC at passage 4 compared to FB. Since DSCC differentially expressed more IP-related genes with significance, for example, the lower expression of* H2db* and* Tap2*, we compared DSCC to FB in subsequent experiments.

At the protein level, Inhba, which inhibits immune responses [19, 20], was 3.4-fold higher in DSCC compared with FB, suggesting IP might be functionally present in DSCC (Figure 1(a)). In addition, we also found increased expression of bone morphogenetic protein 6 (BMP6), which promotes fetal pancreas development, including insulin-producing beta cells [21, 22], and fibroblast growth factor 2 (Fgf2), which is involved in angiogenesis and is a putative factor involved in islet regeneration [23, 24].

We next evaluated the cells' ability to promote allogeneic immune responses in a coculture assay. IFN*γ* was used as a marker for proinflammatory cell activation [14, 15, 25]. A decreased secretion of IFN*γ* was found in the coculture of splenic leukocytes from Balb/c mice plus DSCC from B6 mice after 5 days of incubation, suggesting the capacity to stimulate allogeneic responses was reduced in DSCC compared with FB (Figure 1(b), 25.39 ± 3.09 versus 49.14 ± 8.15 pg/mL, *P* < 0.03, *n* = 4). Furthermore, the percentage of IFN*γ* ^+^ cells in both CD4^+^ and CD8^+^ subsets was also reduced with DSCC coincubation compared with FB (Figure 1(b), CD4^+^IFN*γ* ^+^: 7.64 ± 0.79 versus 18.77 ± 2.98%, *P* < 0.01; CD8^+^IFN*γ* ^+^: 16.22 ± 2.18 versus 28.57 ± 3.77%, *P* < 0.03, *n* = 4), indicating DSCC reduced both allogeneic CD4^+^ and CD8^+^ T cell activation.

This result indicated a potential for DSCC in promoting islet allograft transplantation survival by inhibiting alloreactive T cell activation. We next detected whether DSCC affected islet survival by coculture of DSCC and islets* in vitro* for 3 or 7 days. An increase of insulin (2.1-fold) and a decrease of* Fas* (2.8-fold) and* Bax* (2.2-fold) was detected at the mRNA level in the islets cocultured with DSCC compared with FB, but no statistical significance was found (Figure 1(c)), suggesting some possible beneficial effects of DSCC on islet survival. To further test the effects of DSCC on islet survival, we evaluated beta cell function by static incubation assay. The ability of insulin secretion upon glucose stimulation was significantly improved after 7 days of coculture of isolated islets with DSCC compared with FB (Figure 1(c), 0.29 ± 0.06 versus 0.15 ± 0.05 ng/islet, *P* < 0.01, *n* = 3). These results suggested that the ability of DSCC to preserve beta cell function might be through inhibiting apoptotic genes,* Fas* and* Bax*, expression.

### 3.2. DSCC Prolong Islet Graft Survival in a Fully MHC Mismatched Mouse Islet Transplantation Model

**Figure 2:**
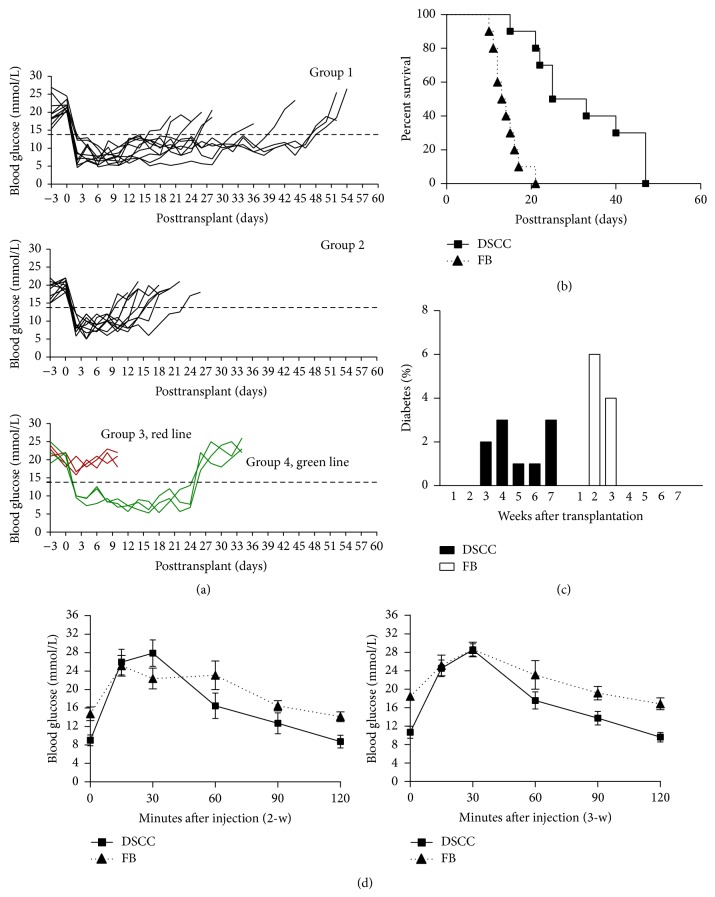
DSCC prolongs mouse islet allograft survival. (a) Blood glucose level in B6 mice before and after transplantation. Diabetes was induced by a single injection of streptozotocin in B6 mice. Mice in groups 1, 2, and 4 returned to normal glucose levels after transplantation with 400 islets from Balb/c donors in a collagen matrix with DSCC (groups 1 and 4) or FB (group 2) cells. Mice in group 3 (DSCC only) did not normalize high glucose after transplantation (red line). (b) Significantly prolonged euglycemia in diabetic B6 mice transplanted with DSCC and islets (black squares) was observed compared to that in mice with FB and islets (black triangles) (*n* = 10, mean 32.2 versus 14.1 days, resp.). Kaplan-Meier graft survival curve is derived from blood glucose data, with *P* < 0.001 by the log-rank test. (c) Distribution of developing graft failure after transplantation is shown in (c). *y*-axis shows the numbers of mice that developed diabetes after transplantation. One week = day 1 to 7; 2 weeks = day 8 to 14; 3 weeks = day 15 to 21; 4 weeks = day 22 to 28; 5 weeks = day 23 to 35; 6 weeks = day 36 to 42; and 7 weeks = day 43 to 49. (d) Intraperitoneal glucose tolerance test results in B6 recipients transplanted with DSCC and islets (*n* = 6) 2 and 3 weeks after transplant (black squares) were compared to the FB and islets transplanted group (*n* = 6) (black triangles). Data are expressed as means ± SE; a better response was found at both time points.

Despite the fact that DSCC express IP genes and have the capacity to inhibit T cell proliferation* in vitro*, there is no direct evidence to show their IP functionality in the context of allogeneic islet transplantation. To investigate the potential local immunosuppressive effect of DSCC, composite grafts of Balb/c derived isolated islets, with DSCC (group 1) or FB (group 2) derived from B6 mice, DSCC only without islets (group 3), and DSCC with islets (group 4, the same as group 1 but nephrectomy was performed 3 weeks after transplant), in a collagen matrix, were transplanted to diabetic immune-competent recipients (B6). Three groups (groups 1, 2, and 4) promptly reversed the blood glucose level of streptozotocin-induced diabetic B6 graft recipients (Figure 2(a)), and group 3 (DSCC only) maintained hyperglycemia, indicating DSCC alone was unable to normalize high glucose levels after transplantation. Mice transplanted with FB and islets (group 2) rejected the allografts around 2 weeks after transplant after establishing primary islet function (Figure 2(a)). In contrast, mice transplanted with DSCC and islets (group 1) showed euglycemia for much longer (Figure 2(b), 32.2 ± 12.2 versus 14.1 ± 3.3 days, *P* < 0.001, *n* = 10). During week 2, 6 out of 10 recipients (60%) developed graft failure in group 2, but none of group 1 did (Figure 2(c)). During week 3, the remaining 4 mice in group 2 (40%) all developed hyperglycemia; 2 out of 10 mice in group 1 (20%) also failed. Of mice in group 1, 3 (30%), 1 (10%), 1 (10%), and 3 (30%) developed graft failure during weeks 4, 5, 6, and 7, respectively, indicating a delay in allograft destruction in the presence of DSCC compared with FB (Figure 2(c)). In addition, it was confirmed that the transplanted DSCC and islets were responsible for this prolonged survival since removal of grafts resulted in high glucose (Figure 2(a), group 4).

To further evaluate beta cell function in the transplanted islets, Intraperitoneal glucose tolerance test (IPGTT) was performed on both groups after 2- and 3-week transplantation (Figure 2(d)). Normal responses of islet grafts to IPGTT demonstrated that DSCC-islets were able to function normally in response to glucose stimulation, suggesting maintenance of islet mass.

### 3.3. DSCC Enhance Angiogenesis

Our observation of delayed allograft rejection and normal response to glucose stimulation in DSCC and islet transplant combinations suggested preservation of beta cell mass. Up to 70% loss of donor islets by isolation and immune rejection in the first week after transplant was found in previous studies [26–28]. Pancreatic islets contain a dense capillary network, approximately 10 times that of surrounding exocrine tissue, for supplying 5 to 10% of the pancreatic blood flow. Similar to islets, HFs also have a high demand for blood supply to ensure hair growth and cycling [27–29]. In fact, HFs have great capacity for stimulating angiogenesis [29–31]. Cotransplantation of islets with HF derived cells that retain their angiogenic properties may be desirable to promote islet vascularization during the early stages of implantation and to help with islet survival. We therefore compared revascularization 1-w posttransplant.

**Figure 3:**
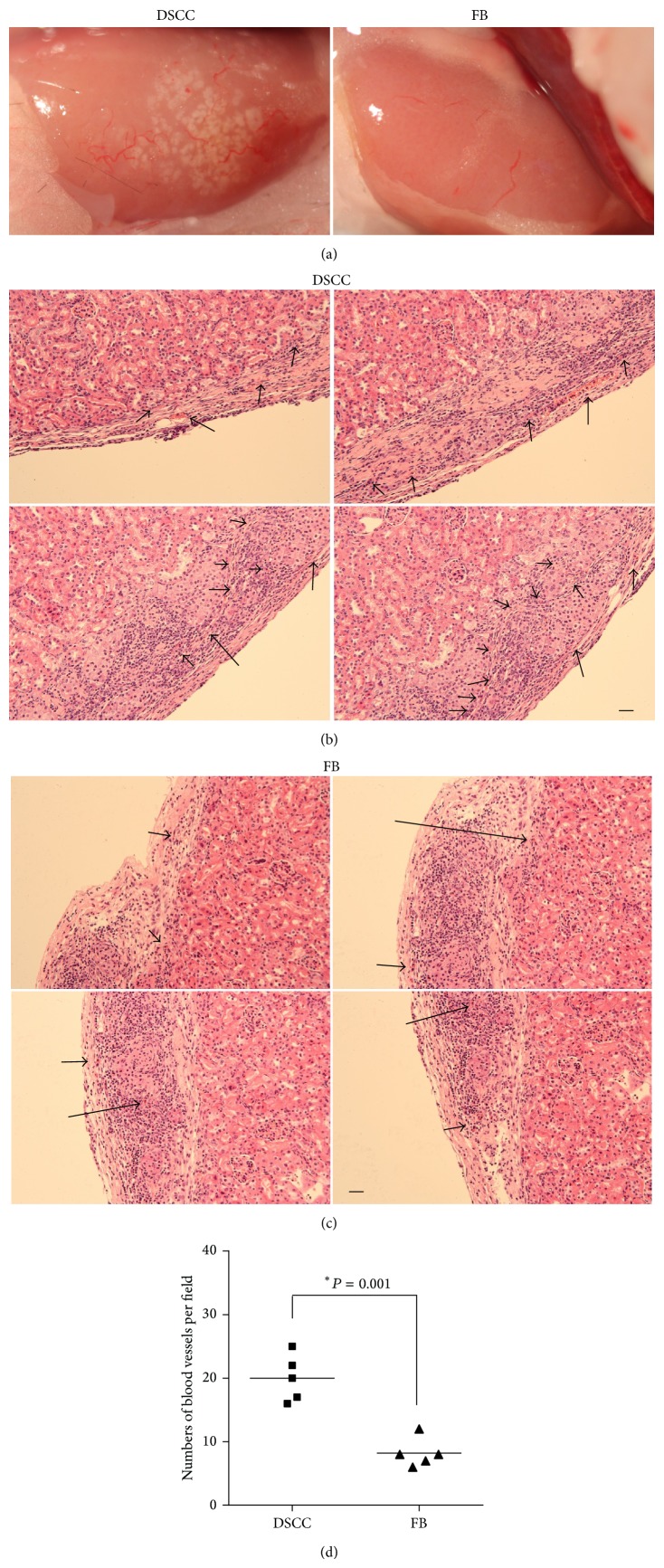
DSCC enhances angiogenesis. (a) Allografts are shown from groups 1 and 2 after 1 week of transplantation. We divided each graft image into 100 grids and counted the numbers of blood vessels in both groups. Higher numbers of blood vessels were observed in group 1 DSCC and islet allografts (left 18 ± 3%) compared with group 2 FB and islet allografts (right 8 ± 2%). Tissue sections of H&E staining are displayed from groups 1 (b) and 2 (c) one week after transplant. ((b) and (c)) Four images in each group consist of panoramic views of the graft. The numbers of big blood vessels (>50 *μ*m diameter) were more in group 1 than in group 2 (4 ± 2% versus 1 ± 1%). Black arrows indicate the blood vessels. (d) Quantification of the blood vessels. Scale bar = 50 *μ*m.

A higher density of infiltrating blood vessels was noticed in the allografts of group 1 (DSCC) mice compared with group 2 (FB, Figure 3(a), 17.33 ± 3.05 versus 6.67 ± 3.06%, *P* < 0.01, *n* = 3) mice. Furthermore, overall size of the blood vessels was bigger in group 1 than in group 2. This was reflected by the numbers of the blood vessels larger than 50 *μ*m in diameter being significantly higher in group 1 compared with group 2 (Figures 3(b) and 3(c), 4.00 ± 1.00 versus 0.67 ± 0.57%, *P* < 0.01, *n* = 3) per field. In addition, the total numbers of the blood vessels were significantly higher in group 1 (Figure 3(d)). The location of the blood vessels is also closer to the transplanted islets in group 1 (Figure 3(b)). In summary, the higher numbers and larger size of the blood vessels in group 1 compared with group 2 demonstrate an enhanced angiogenesis in the presence of DSCC.

### 3.4. DSCC Modulates Immune Responses in the Draining Lymph Nodes of Transplanted Mice

**Figure 4:**
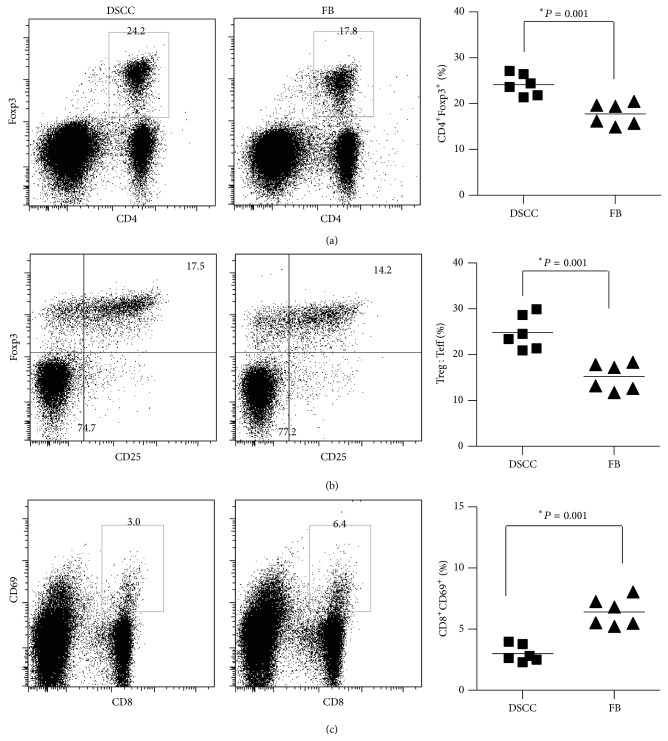
DSCC modulates immune responses in the draining lymph nodes of transplanted mice. Representative dot plots (left) and accumulating data (right) of CD4^+^Foxp3^+^ (a), CD25^+^Foxp3^+^ (b), and CD8^+^CD69^+^ (c) cells from the renal lymph nodes of transplanted mice are shown. Each dot represents the result from one mouse in group 1 (DSCC and islets) or 2 (FB and islets) after 2 weeks (a, b) or 3 weeks (c) of transplantation. Pooled data (right) are shown as the means from six mice per experiment, with Student *t*-test showing significance, ^∗^ *P* < 0.05.

DSCC stimulated lower alloimmune responses* in vitro* compared with FB. We next investigated the effects of DSCC on alloimmunity* in vivo* by examining T cell activation markers CD25, CD69, and CD44 and T cell regulatory marker Foxp3. We observed a significant increase of CD4^+^Foxp3^+^ (Figure 4(a), 24.16 ± 0.96 versus 17.81 ± 0.98%, *P* < 0.001, *n* = 6) and CD25^+^Foxp3^+^ (Figure 4(b), 24.81 ± 1.53 versus 15.20 ± 1.20%, *P* < 0.001, *n* = 6) and a decrease of CD8^+^CD69^+^ (Figure 4(c), 3.00 ± 0.29 versus 6.41 ± 0.47%, *P* < 0.001, *n* = 6) cells in the presence of DSCC compared with FB in the renal lymph nodes of transplanted mice after one week (Figures 4(a) and 4(b)) and two weeks (Figure 4(c)), respectively. However, no significant changes were found at three weeks or in the spleens at any time point (data not shown). These results indicated DSCC did not change the systemic immune profiling in the spleen; instead they locally modulated regulatory T cells (Figures 4(a) and 4(b)) at one week, suggesting the capacity of DSCC to inhibit alloreactive T cell activation partially through upregulating inhibitory T cell subsets. Indeed, reduced T cell activation was detected at two weeks (Figure 4(c)), demonstrating that DSCC also stimulated reduced alloimmune responses* in vivo*.

### 3.5. DSCC Prevents Infiltration of Leukocytes into the Allograft

**Figure 5:**
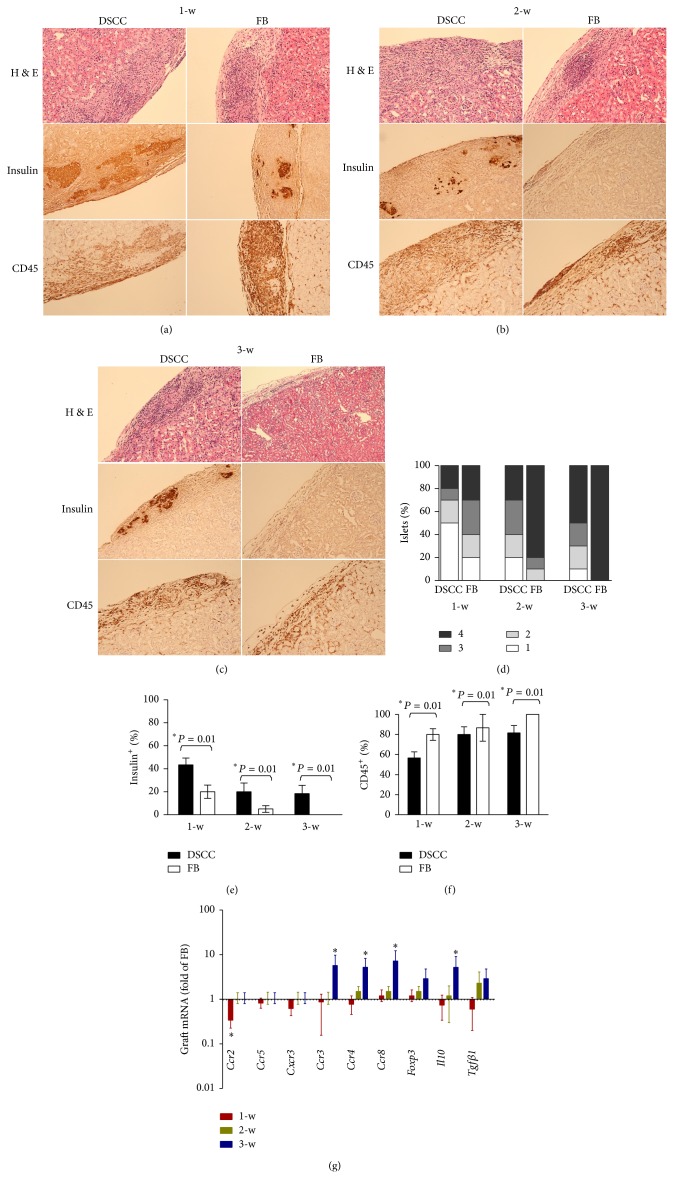
DSCC partially prevents infiltration of leukocytes into the allografts. Representative tissue sections of transplanted grafts of group 1 (DSCC and islets) or group 2 (FB and islets) after one week (a), two weeks (b), or three weeks (c) are presented. The insulin^+^ or CD45^+^ cells are stained brown (DAB system). Isletitis grade (d) was assessed as described previously [16]. Grade 1, mononuclear infiltration, largely in the periphery, in less than 25% of the islets; grade 2, 25 to 50% of islets showing mononuclear infiltration; grade 3, over 50% of islets showing mononuclear infiltration; and grade 4, over 75%, small, retracted islets with few mononuclear cells. (e) Numbers of insulin^+^ stained cells were calculated in three individual allografts per field. A significantly higher number of insulin^+^ cells were in group 1 (DSCC) transplants compared with group 2 (FB) transplants at all three time points. No insulin^+^ cells were found in group 2 transplants at three weeks after transplant. (f) Numbers of CD45^+^ stained cells were calculated in three individual allografts per group. (g) Kinetics of mRNA expression for Th1 (*Ccr2*,* Ccr5*, and* Cxcr3*) chemokines, Th2 (*Ccr3*,* Ccr4*, and* Ccr8*) chemokines,* Foxp3*,* Il10*, and* Tgfβ1* was detected by quantitative real-time PCR in the allografts from group 1 (DSCC) and group 2 (FB) after one week (in red), two weeks (in green), and three weeks (in blue) of transplantation. (d), (e), and (f) were generated from 3 individual mice per group. (g) represents the results from three different mice with duplicated repeats, with Student *t*-test showing significance, ^∗^ *P* < 0.05.

Rejection of transplanted grafts involves destruction of insulin-producing beta cells by leukocytes that infiltrate into the islets. A reduction of graft failure in group 1 (DSCC, Figures 2(a) and 2(b)) may be due to reduced graft inflammatory cell infiltration. Therefore, the development of insulitis, insulin^+^ cells (beta cell function), and CD45^+^ cells (infiltrating leukocytes) were examined in group 1 (DSCC) and group 2 (FB) (Figures 5(a)–5(f)). Insulitis was detected in both groups 1 and 2. However, a significantly reduced insulitis was observed in group 1 compared with group 2 at all three time points (Figure 5(d), 1-w, 2-w, and 3-w), suggesting reduced inflammation in the presence of DSCC.

We observed a significant loss of insulin^+^ cells (more than 50%) 1-w after transplantation in both groups (Figure 5(e), 43.33 ± 10.41 at 1-w versus 20.00 ± 13.23 at 2-w, 53.84% of loss in group 1; 20.00 ± 10.00 at 1-w versus 5.00 ± 5.00 at 2-w, 75.00% of loss in group 2), suggesting severe destruction of transplanted islets by alloreactive leukocytes or other nonimmune factors. However, numbers of insulin^+^ cells were higher in group 1 compared with group 2 at all three time points, suggesting less damage of beta cells in the presence of DSCC. The better preservation of beta cell function in group 1 may be the result of fewer infiltrating inflammatory (CD45^+^) cells. Indeed, significantly lower CD45^+^ cell numbers were found in group 1 compared with group 2 (Figure 5(f), 56.67 ± 10.41 versus 80.00 ± 10.00 at 1-w; 80.00 ± 13.23 versus 86.67 ± 23.09 at 2-w; 81.67 ± 12.58 versus 100 at 3-w, *P* < 0.01), suggesting better control of infiltration in the presence of DSCC.

To further investigate the mechanisms of DSCC on islet transplantation, we examined the expression profile of selected chemokines and potent immunosuppressive factors in the transplanted grafts. Quantitative PCR showed a significant decrease of* Ccr2* (Th1 chemokine) at 1-w and an increase of* Ccr3*,* Ccr4*,* Ccr8* (Th2 chemokine), and* Il10* (potent immunosuppressive factor) at 3-w in group 1 (DSCC) compared with FB (Figure 5(g)). Thus, promoting a Th2 immune response shift and creating an immunosuppressive microenvironment in the later stages (3-w) by inhibiting Th1 proinflammatory chemokines in early stages (1-w) may be one of the reasons for more limited leukocyte infiltration into DSCC-islet grafts.

### 3.6. DSCC Protects Transplanted Islets without Systemic Immunosuppression

**Figure 6:**
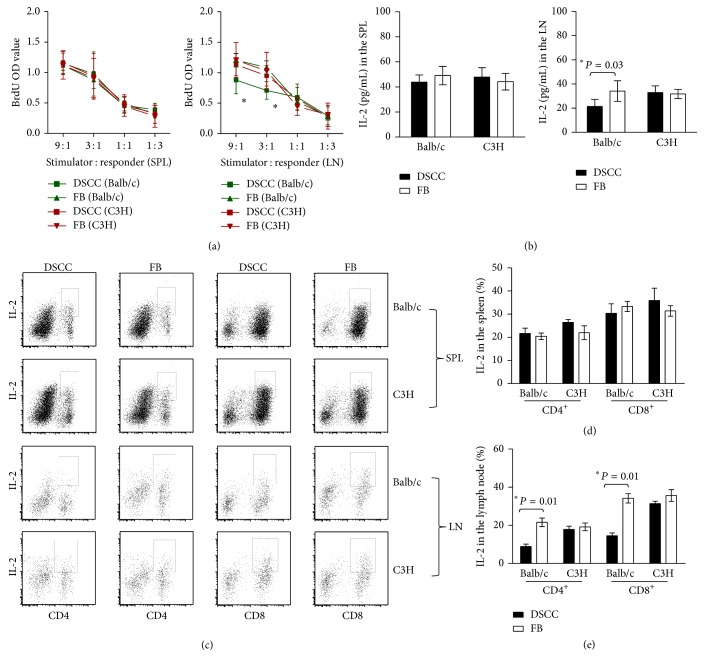
DSCC protects transplanted islets without systemic immunosuppression. (a) Allogenic mixed-lymphocyte reaction (MLR) type assays of leukocytes from group 1 (DSCC) mice were compared with leukocytes from group 2 (FB) mice at week two after transplantation. The responses of the responders from group 1 (DSCC, square) and those of responders from group 2 (FB, triangle) B6 (H-2b) recipients to stimulator *γ*-irradiated-splenocytes (at 2500 rad) from Balb/c (left, H-2d) or C3H (right, H-2k, third party control) were tested at the indicated ratios after 4 days of incubation. Wells were pulsed with BrdU for the last day, and BrdU incorporation into DNA was measured in triplicates. There was no significant difference in the leukocyte responders from the spleen (left) or renal lymph nodes (right) of group 1 and group 2 mice in response to C3H derived splenocyte cell stimulation. ^∗^Significant differences between groups 1 and 2 renal lymph node leukocytes, but not splenic leukocytes, at stimulator/responder ratios of 9 : 1 (*P* < 0.04) and 3 : 1 (*P* < 0.01, *n* = 6) were noticed in response to Balb/c derived splenocyte stimulation. (b) The total amount of IL-2 secretion in the MLR culture was detected by ELISA and expressed in pg/mL. No significantly different IL-2 secretion was observed in response to C3H derived splenocyte cell stimulation in the two groups. Significant differences in IL-2 secretion were observed between groups 1 and 2 renal lymph node derived leukocyte cell cultures, but no difference was observed in spleen derived leukocytes in response to Balb/c derived splenocyte stimulation. (c) Representative dot plots of IL-2 production in CD4^+^ (left) or CD8^+^ (right) cells are presented from flow cytometric analysis. Accumulating data were calculated based on 3 independent experiments of 6 mice in total per group. (e) A significant decrease of IL-2 expression in both CD4^+^ and CD8^+^ subsets from renal lymph nodes from group 1 (DSCC) compared with group 2 (FB) was noticed in response to Balb/c but not C3H derived splenocytes. (d) No significant difference was identified in the spleen cells in response to either Balb/c or C3H splenocyte stimulation. Each dot represents one mouse. ^∗^ *P* < 0.05.

More limited infiltration of leukocytes (Figure 5) in the allografts from group 1 (DSCC) may result from impaired immunity to alloantigens. To investigate this possibility, MLRs were performed. Results showed similar BrdU incorporation into spleen (SPL) cells in response to either alloantigenic Balb/c or third party control (C3H) splenic leukocytes, suggesting unchanged systemic immune responses in both groups (Figure 6(a)). However, reduced BrdU incorporation was found in the renal lymph node (LN) cells from group 1 (DSCC) mice in response to Balb/c cells but not C3H cells, indicating donor-specific hyporesponsiveness in the local draining lymph nodes (Figure 6(a), 9 : 1 ratio: 0.87 ± 0.21 versus 1.21 ± 0.27, *P* < 0.04; 3 : 1 ratio: 0.71 ± 0.14 versus 1.08 ± 0.27, *P* < 0.01, *n* = 6). This result was confirmed by differences in the total amount of IL-2 secretion (Figure 6(b), 21.34 ± 3.34 versus 33.83 ± 8.87 pg/mL, *P* < 0.03). Furthermore, the percentage of IL-2 in both CD4^+^ and CD8^+^ subsets in the LN, but not in the SPL in group 1, was lower than that in group 2, demonstrating impaired alloreactive CD4^+^ and CD8^+^ T cells in the local LN but not systemically (Figures 6(c), 6(d), and 6(e), CD4^+^IL-2^+^: 8.84 ± 2.57 versus 21.58 ± 4.45, *P* < 0.01; CD8^+^IL-2^+^: 14.53 ± 2.93 versus 34.16 ± 4.90, *P* < 0.01; *n* = 6).

## 4. Discussion

Prevention of allograft rejection can be achieved by either systemic or local immunosuppression [1, 16, 17]. Although local immunosuppression controls islet rejection successfully, most studies use gene transfer approaches, which potentially have deleterious effects in human transplantation [16, 17]. Moreover, genetically modified cells typically rely on just one IP conferring product. The use of nongenetically modified cells with natural IP avoids these issues. The concept of controlling alloreactive T cell responses by naturally immune privileged cell therapy has been investigated in several experimental models including islet cell transplantation using amniotic or Sertoli cells [5–8].

In the current study, we showed that cotransplantation of naturally immune privileged HF dermal sheath cup cells (DSCC) with donor islets prolonged allograft survival without systemic antirejection treatments in diabetic immune-competent mice. Consistent with HF IP properties, we showed that cultured DSCC also expressed lower levels of MHC I and related gene* Tap2* and a higher level of potent immunosuppressive protein Inhba, compared with non-IP tissue derived FB. The potential of IP status in cultured DSCC was confirmed by both* in vitro* coculture assay and a fully MHC mismatched mouse islet transplantation model. Several lines of evidence showed that cultured DSCC possessed the capacity to limit alloimmune T cell responses. We first demonstrated DSCC stimulated reduced alloreactive T cell activation using IFN*γ* as a surrogate maker in an* in vitro* coculture functional assay. We also showed that DSCC maintained lower CD8^+^ activation in the local renal draining lymph nodes and attracted a more limited infiltration of leukocytes in MHC mismatched islet allografts.

The transient increase of regulatory T cells in our* in vivo* model may explain the ability of DSCC to control alloreactive T cell activation. Tregs have been implicated in promoting and maintaining IP status [32–35]. Tregs play a critical role in successful transplantation of corneal tissue which also has IP [35]. Our data provide another line of evidence for the role of Tregs in preserving beta cell function and promoting allograft survival. In addition, a Th1/Th2 shift is also important in determining the outcomes of transplantation and beta cell survival [16, 36]. Our data show that DSCC promote an anti-inflammatory microenvironment in the allograft and shift the Thl/Th2 balance toward Th2.

Our findings suggest significant revascularization of transplants in the presence of DSCC. The rationale behind this finding is closely related to normal HF function. The HF is a mini organ with great regenerative potential since humans are constantly in a state of growing and losing hair. Hair cycling affects blood vessel arrangement around the HF itself and surrounding skin vascularization [29–31]. Encouraging new blood vessel formation could be beneficial for providing essential nutrients and oxygen for beta cell survival since a significant number of donor islets are lost in the early stages after transplant due to poor revascularization and lack of blood supply associated with the isolation and transplantation processes [26–28]. This finding supports the notion of DSCC preserving beta cell mass in multiple ways.

This study for the first time shows that DSCC generate donor-specific tolerance. More importantly, B6 mice implanted with DSCC responded to third party C3H cell stimulation normally, excluding the possibility of nonspecific immune suppression or immune ignorance. Tolerance occurs through various mechanisms including anergy/ignorance and immune regulation/suppression [37–39]. It is well documented that Tregs actively induce tolerance. Our finding of transient upregulation of Tregs suggests their potential involvement in promoting tolerance to the islet grafts. The detailed mechanism underlying this finding is currently under investigation.

Although the idea of using naturally IP DSCC in protection of transplanted islets for treating autoimmune diabetes is promising, more questions need to be answered. For example, different cell numbers and generations can be used for cotransplantation to optimize success rates. Genetic loss or gain of function modification approaches could be employed to examine the relative significance of individual IP conferring factors we have identified in the current study. The finite survival of donor islets in the recipients may appear discouraging at first glance. However, data presented here show that localized, but not systemic, immunosuppression was achieved. Better outcomes may be obtained with more robust, extensive investigations. At a minimum, a DSCC cotransplantation technique could be used with immunosuppressive drug regimens at reduced dose to improve graft survival and eliminate or reduce undesirable side effects.

In conclusion, we demonstrate proof-of-principle in using cultured HF derived cells to create localized immunosuppression without using antirejection drug agents to protect islet allografts as a new potential therapeutic treatment for autoimmune diabetes. DSCC reduce islet allograft rejection through promoting new blood vessel formation and better beta cell survival and limiting alloreactive T cell attack. This result opens new avenues for using cells with natural IP in treating diabetes.

## Figures and Tables

**Table 1:** PCR primer pairs.

Gene	Sense	Antisense
*Bax *	TGC AGA GGA TGA TTG CTG AC	GAT CAG CTC GGG CAC TTT AG
*Bmp6 *	AAT GAC GAC GAA GAG GAT GG	AGA CTC TTG CGG TTC AAG GA
*Ccr2 *	AGA GAG CTG CAG CAA AAA GG	GGA AAG AGG CAG TTG CAA AG
*Ccr3 *	GAT TGC CTA CAC CCA CTG CT	TGC CAC ATT TCT GTG GAA AA
*Ccr4 *	AGG CAA GGA CCC TGA CCT AT	GCA GTA CGT GTG GTT GTG CT
*Ccr5 *	CGA AAA CAC ATG GTC AAA CG	TTC CTA CTC CCA AGC TGC AT
*Ccr8 *	GCA GTC TTT GAG GTG GAA GC	GAT GGC TCT GGT CCT GTT GT
*Cxcr3 *	ATG CCT TTG TGG GAG TGA AG	AGG AGG CCT CAG TTG TCT CA
*Fas *	TGC TTG CTG GCT CAC AGT TA	CAT GGT TGA CAG CAA AAT GG
*Fgf2 *	AGC GGC TCT ACT GCA AGA AC	TGG CAC ACA CTC CCT TGA TA
*Foxp3 *	TTC ATG CAT CAG CTC TCC AC	CTG GAC ACC CAT TCC AGA CT
*H2db *	ATG GAG CTT GTG GAG ACC AG	CAC GGC ATG TGT AAT TCT GC
*Il1ra *	TTG TGC CAA GTC TGG AGA TG	TTC TCA GAG CGG ATG AAG GT
*Il10 *	CCA AGC CTT ATC GGA AAT GA	TTT TCA CAG GGG AGA AAT CG
*Inhba *	CGG AAG AGT ACC TGG CAC AT	GCC CAG AAG CAC TAG ACT GG
*Ins *	GGA GCG TGG CTT CTT CTA CA	GTG CAG CAC TGA TCC ACA AT
*Tap2 *	AAG GTG GTG GGG CTC TA CTT	GGG GGT TGT ACA CCT TCT CA
*Tgfβ1 *	TTG CTT CAG CTC CAC AGA GA	TGG TTG TAG AGG GCA AGG AC
*Vegfr1 *	TGA GGA GCT TTC ACC GAA CT	TAT CTT CAT GGA GGC CTT GG

## Abbreviations

ELISA: Enzyme-linked immunosorbent assay
HF: Hair follicle
IFN*γ*: Interferon-gamma
IP: Immune privilege
MHC I, II: Major histocompatibility complexes I, II
PBMC: Peripheral blood mononuclear cells
qPCR: Quantitative real-time RT-PCR.
